# Von Willebrand disease: more than just a bleeding disorder

**DOI:** 10.1016/j.rpth.2025.103276

**Published:** 2025-12-05

**Authors:** Dawn Swan, Michelle Sholzberg, Bethany Samuelson Bannow, Jecko Thachil

**Affiliations:** 1 Department of Haematology, Austin Health, Melbourne, Victoria, Melbourne, Australia; 2 Departments of Medicine, Laboratory Medicine, and Pathobiology, St. Michael’s Hospital, Li Ka Shing Knowledge Institute, University of Toronto, Toronto, Ontario, Canada; 3 Division of Classical Haematology, Department of Haematology/Oncology, Cleveland Clinic Foundation, Cleveland, Ohio, USA; 4 Department of Haematology, Manchester Academic Health Science Centre, University of Manchester, Manchester, United Kingdom

**Keywords:** heavy menstrual bleeding, psychosocial, socioeconomic, von Willebrand

## Abstract

Von Willebrand disease (VWD) is the most common inherited bleeding disorder in the world. It is characterized by mucocutaneous bleeding, with heavy menstrual bleeding affecting the vast majority of girls and women with this condition. However, while bleeding episodes are the hallmark of VWD, there are marked psychological and socioeconomic ramifications of this chronic disorder, which may impact patients throughout their lifetimes, as well as additional medical issues, which may affect women with VWD preferentially. In this review, we discuss the evidence in support of nonbleeding complications of VWD and the key importance of providing a holistic approach to patient care.

## Essentials

• Nonbleeding manifestations of VWD are overlooked.
• Literature search for the nonbleeding manifestations of VWD was undertaken.
• Reproductive issues, musculoskeletal complications, and other medical problems are highlighted.
• Psychosocial impact and socioeconomic consequences are also addressed.

## 1 Introduction

Von Willebrand disease (VWD), initially described by Finnish physician Erik von Willebrand in 1926, is the most common inherited bleeding disorder in the world [1], affecting up to 1% of the general population [2]. It is classified into 3 types and several subtypes [3]. While VWD is inherited equally between men and women, type 1 VWD is diagnosed more frequently in women, with twice the reported incidence (4.8 vs 2.4/100,000 people). This is thought to be due to presentation to medical attention for heavy menstrual bleeding (HMB) or postpartum hemorrhage (PPH) [4].

VWD is characterized by mucocutaneous bleeding. HMB is the most common type of bleeding and is reported to affect 60% to 95% of girls and women with VWD [5,6]. In fact, of individuals in the general population presenting with HMB, 5% to 20% are subsequently found to have VWD [[7], [8], [9]]. Other common bleeding symptoms include gastrointestinal bleeding, epistaxis, bruising, and bleeding related to trauma or surgery [10]. Hemarthroses occur in approximately 10% to 25% of patients, most typically those with type 3 disease [[10], [11], [12], [13]]. Gastrointestinal bleeding accounted for around 13% of major bleeding episodes in a study of 44 patients with VWD receiving plasma-derived von Willebrand factor (VWF):FVIII concentrate [14].

## 2 Beyond Bleeding in Women with VWD

While HMB represents the most common symptom experienced by women with VWD, they may also be at an increased risk of other health conditions, such as endometriosis, endometrial hyperplasia and polyps, miscarriage, migraine, iron deficiency (ID), arthropathy, and poor bone health. In this study, we review a list of conditions for which patients with VWD are at increased risk, distinct from clinically apparent bleeding. While some may be downstream consequences of missed or untreated bleeding, the pathophysiological link to others remains unknown. The informed clinician will remain vigilant for these associated conditions and screen and treat, or refer, accordingly.

**Figure 1:**
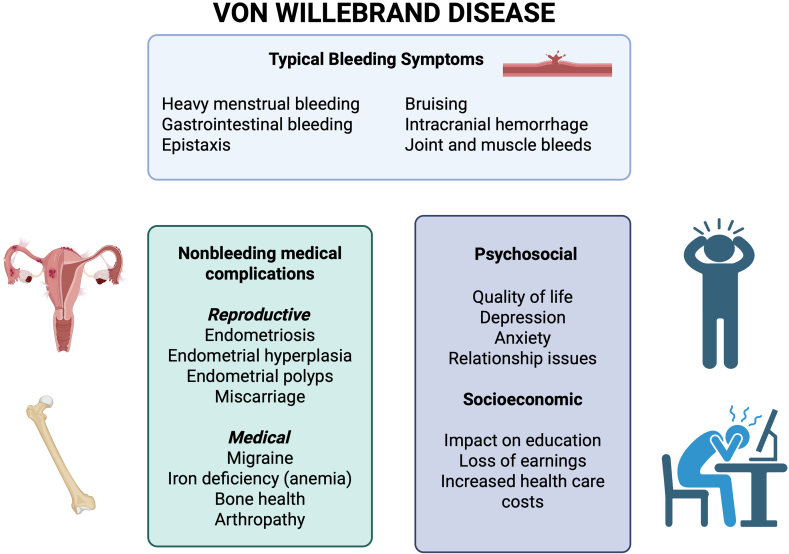
The overall impact of von Willebrand disease on women and girls: bleeding and nonbleeding manifestations, as well as psychosocial and socioeconomic implications.

A summary of the quality of the supportive evidence demonstrating an association between VWD and these conditions is shown in Table 1 [[15], [16], [17], [18]], and an overview of the topics presented is provided in Figure 1.

**Table 1:** Level of evidence present for von Willebrand disease complications.

Complication	Possible pathophysiological mechanisms	Compelling evidence	Mixed evidence	Studies needed
Endometriosis	Increased retrograde menstruation [15]			X
Endometrial hyperplasia and polyps	Unknown/possible reporting bias			X
Miscarriage	Unknown/possible reporting bias		X	
Migraine	Unknown/possible reporting bias			X
Iron deficiency	Increased bleeding [16]	X		
Arthropathy	Hemarthroses [17]	X		
Bone health	Interaction between VWF and OPG/RANK/RANKL [18]	X		
Reduced quality of life		X		
Psychosocial issues		X		

X merely indicates the selection made.

OPG, Osteoprotegerin; RANK, receptor activator of nuclear factor κB; RANKL, receptor activator of nuclear factor κB ligand; VWF, von Willebrand factor.

## 3 Reproductive Health Complications

### 3.1 Endometriosis

Endometriosis, a condition characterized by the presence of endometrial tissue outside of the uterus, is associated with dysmenorrhea, pelvic pain, and impaired fertility [19]. It is reported to affect around 5% to 10% of women, although delayed diagnosis is common, and rates may be higher [20,21]. In a single-case-controlled study by the US Centers for Disease Control and Prevention (CDC) in 2003, the authors conducted telephone interviews on 102 women with VWD of variable severity registered in hemophilia treatment centers and compared their responses with 88 controls, to assess incidence of a variety of conditions [22]. In this study, the incidence of endometriosis was significantly higher among patients with VWD compared with controls (30% vs 13%; *P* = .005) [22]. A number of case reports in the literature also describe unusual or recurrent cases of endometriosis in patients with VWD, but further larger studies are lacking [21,23].

The exact pathogenesis of endometriosis remains an area of study; however, the concept of retrograde menstruation is the most widely accepted. This theory proposes that menstrual blood is refluxed from the uterine cavity into the abdominal cavity via the fallopian tubes during menstruation and forms abnormal deposits. As women with VWD have heavier menstrual blood loss, their risk of retrograde menstruation and subsequent endometriosis may be higher [15]. In addition, women with VWD are likely to have increased bleeding from extrauterine endometrial deposits, and the associated pain may translate to a higher rate of presentation to medical services and subsequent diagnosis [24].

Conversely, endometriosis is associated with chronic inflammation, with increased serum levels of fibrinogen, D-dimer, and plasminogen activator inhibitor 1 detected compared with healthy controls [25]. A Mendelian randomization study using 12,642 cases of VWD and 286,469 controls from the UK Biobank and FinnGen European ancestry cohort reported a reduced risk of endometriosis in individuals predicted to have higher levels of the metalloproteinase ADAMST-13, and thus lower levels of high-molecular-weight VWF multimers, based upon genetic markers, However, genetically predicted higher VWF plasma levels were associated with an increased risk of endometriosis [26]. Further study is required to determine the pathogenesis of this condition and how this impacts patients with VWD.

### 3.2 Endometrial hyperplasia and polyps

Endometrial hyperplasia is a heterogeneous condition ranging from benign to premalignant proliferation of the uterine lining. It is caused by the effect of unopposed estrogen, when estrogen is not balanced by a sufficient amount of progesterone, and is most commonly seen around or postmenopause [27]. The CDC study by Kirtava et al. [22] showed that 10% of patients with VWD reported a diagnosis of endometrial hyperplasia compared with 1% of controls (*P* = .01) [22]. There is no other published literature describing an association with VWD, so this may represent increased diagnosis in patients more likely to present to medical services due to bleeding. This may potentially be exacerbated by reporting bias, in that patients with diagnosed VWD may have greater health literacy than some controls, leading to increased awareness of medical conditions and a higher rate of reporting [24]. Similarly, uterine polyps were present in 8% of VWD cases compared with 1% of controls (*P* = 0.04) [22], but this also likely represents diagnostic bias.

### 3.3 Miscarriage

Kirtava et al. [22] reported an increased rate of miscarriage in their study of 102 women with VWD compared with 88 healthy controls (15% pregnancies ended in miscarriage vs 9%; *P* = .05) [22]. While they identified more miscarriages in the VWD cohort, the routinely accepted rate of miscarriage in the general population is also 15% [28]. A number of case series have also reported higher frequencies of miscarriage. A retrospective analysis of 31 patients with VWD registered at the Royal Free Haemophilia Centre in London from 1980 to 1996 reported a miscarriage rate of 22%, with threatened miscarriage in one-third of patients [8]. Similarly, in an International Society of Haemostasis and Thrombosis (ISTH) VWD registry, 69 pregnancies were reported in 31 women. Miscarriage occurred in 22% (15 pregnancies); however two-thirds of these were in 4 women [29]. Another larger retrospective study compared 150 women with VWD with 145 with evidence of increased mucocutaneous bleeding, but VWF levels ≥ 0.5 IU/mL and 137 healthy controls. Rates of miscarriage were not statistically different between the groups at 45.3%, 56.6%, and 37.2% respectively, although in this study, the rate of miscarriage in the control arm was higher than that expected [30]. Similar to the ISTH registry report, a number of patients in the VWD and non-VWD bleeding groups experienced recurrent miscarriages. In this study, patients in both bleeding disorder cohorts were more likely to experience recurrent miscarriages than those in the control group (≥2 pregnancy losses in 10.7%, 10.3% and 5.8% of VWD, non-VWD bleeding, and control patients, respectively, and ≥3 losses in 8%, 8.3%, and 2.9%, respectively). These rates were higher than those expected in the general population in all cohorts analyzed (1.9% for ≥2 losses and 0.7% for ≥3 losses) [28,31], which the authors postulated might reflect the questionnaires used in the study [30].

Although rates of miscarriage appear to be higher in individuals with VWD compared with the general population, this may be attributable to increased detection related to bleeding, as a significant proportion of miscarriages usually go undetected. A study conducted in healthy women who were trying to conceive used daily urinalysis to detect early pregnancy losses. There were 198 pregnancies, with a total rate of miscarriage of 31%, including 22% that occurred prior to a clinical diagnosis of pregnancy, and which would not routinely be expected to be identified [32]. Whether VWD is truly associated with a higher risk of miscarriage or whether this simply reflects a greater chance of diagnosis in patients with a mucocutaneous bleeding disorder requires further investigation.

## 4 Medical and Musculoskeletal Complications

### 4.1 Iron deficiency

The high incidence of HMB in women with VWD alongside other bleeding manifestations is associated with extremely high rates of ID. Iron studies were performed in 741 female patients in hemophilia treatment centers in the United States, of which 477 had evidence of ID (defined as a serum ferritin of < 50 μg/L) and 264 did not. Of those with ID, 49.5% had VWD; however, the greatest risk was seen in patients with platelet disorders [16]. A population-based study from Sweden included 136 women aged 18 to 55 years with VWD. ID (ferritin < 30 μg/L) was identified in 45% of the cohort and 18% had iron deficiency anemia (IDA) with a hemoglobin level of < 120 g/L [33]. Identifying and treating ID, with or without associated anemia, is important to mitigate its effects on energy levels, fatigue, cardiovascular health, and cognition [[34], [35], [36], [37]].

Anemia during pregnancy is linked to adverse fetal outcomes such as poor growth, premature delivery, and developmental delay [[38], [39]]. Furthermore, there is a reciprocal connection between ID/IDA and bleeding in pregnancy. In a meta-analysis of 327 studies of PPH, anemia was identified as a strong risk factor for PPH (odds ratio, 2.36; 95% CI, 1.29-4.32) [39]. Impaired transport of hemoglobin and oxygen to the uterus in women with anemia from prior untreated HMB may lead to weakened uterine contractility and a propensity toward atony. Additionally, there may be a compensatory increase in placental vascularity in anemia, further increasing bleeding risk [40,41]. Prenatal anemia is associated with mortality from PPH [42], and clearly, PPH confers a risk of causing or worsening pre-existing anemia.

Although ID is caused by bleeding, it has a personal and intergenerational health impact that extends beyond the issue of bleeding alone and is a distinct condition that occurs in individuals with and without bleeding disorders. Managing ID before the onset of associated morbidity is of paramount importance in women with VWD and requires a unique treatment approach with appropriate iron supplementation alongside hemostatic therapies and/or hormonal approaches in HMB. It is therefore worthy of discussion outside of the context of bleeding symptoms.

### 4.2 Migraine

A number of studies have demonstrated increased VWF antigen levels and activity in patients with migraine compared with healthy controls, prompting the idea that migraine may be less frequent in patients with VWD [43,44]. However, paradoxically, the CDC study by Kirtava et al. [22] actually noted an increased incidence of migraine in women with VWD compared with that in healthy controls (41% vs 13%; *P* < .001) [22], with an incidence of 18% reported from a study of approximately 30,000 members of the general population in the United States [45]. Altered platelet serotonin release, which has been demonstrated in some forms of VWD, particularly platelet-type VWD, is one proposed mechanism [46]. Migraine is also known to be more common among women than men. Fluctuations in estrogen levels likely play a role, as estrogen impacts production of nitric oxide, a key regulator of vascular tone. However, the impact of hormonal changes is modulated by other factors such as age, menopausal status, and use of hormone replacement and varies from patient to patient [47,48].

Another study of 75 patients with VWD, of whom 83% were female, reported an extremely high rate of migraine, in 57% of patients (60% in female participants, 46% in males), interfering with plans in 25% and leading to impaired activity in 15% [49]. However, other studies have identified more modest associations [50]. A number of retrospective studies have also suggested a potential association between ID and IDA and migraine in women [51,52], which may be an important confounding factor in this case. Additionally, the potential impact of VWD treatment has not been specifically explored. Desmopressin, which is commonly used in the management of VWD, causes vasodilation. Headache is a common side effect, but an association with migraine has not been determined [53]. Similarly, headache is also a reported side effect of tranexamic acid, which is commonly used in the management of HMB and mild bleeding symptoms in VWD [54].

### 4.3 Bone health

An association between low bone mineral density (BMD) and hemophilia in males is well established. Risk factors include reduced mobility related to joint bleeds [55,56]; however, the bleeding disorder itself may contribute through a reduction in FVIII-VWF-mediated inhibition of receptor activator of nuclear factor κB (RANK) ligand-induced osteoclastogenesis [18,57]. The RANK/RANK ligand pathway plays a key role in regulating bone turnover. Binding of RANK ligand to RANK on osteoclast precursors stimulates their differentiation and activation. Osteoprotegerin (OPG) is a RANK ligand decoy, produced by osteoblasts, which inhibits this interaction [58]. OPG binds to the A1 domain of VWF within the FVIII-VWF complex, leading to enhanced OPG-RANK ligand binding and downstream inhibition of osteoclast activation [18]. Reduced FVIII-VWD-OPG binding results in greater osteoclastic activity and bone resorption, which has been demonstrated in patients with deficiencies of FVIII and/or VWF [57].

A study of 4546 people including 3555 with VWD (median age of 30 years), compared bone health with `69,900 healthy controls, with a median follow-up of 2.3 years. Patients with VWD were at a significantly higher risk of fractures (incidence rate ratio [IRR], 1.52; 95% CI, 1.07-2.15), need for joint replacement (IRR, 2.02; 95% CI, 1.71-2.37), osteoporosis (IRR, 2.52; 95% CI, 1.91-3.31), and osteopenia (IRR, 2.11; 95% CI, 1.72-2.59) than controls [59]. Another recent study compared 19,580 patients with VWD (of which 14,480 were females) with 71,133,690 controls (38,303,300 females). Rates of osteoporosis, osteoarthritis, and fractures were reviewed from 1999 to 2020 and found to all be significantly higher in female participants with VWD than those in controls (relative risk [RR] of osteoporosis, 1.8; 95% CI, 1.7-1.94; RR of osteoarthritis, 2.0; 95% CI, 2.02-2.15; RR of fractures, 2.0; 95% CI, 1.91-2.09), with higher rates also seen in males with VWD. Hypothyroidism and use of corticosteroids and nonsteroidal anti-inflammatories were identified as risk factors [60].

Patients with VWD appear to be at increased risk of low BMD and associated sequelae. This does not appear to be connected with hemarthroses and impaired mobility in these patients but rather is suggestive of a disease-mechanistic impact on bone health. Given the morbidity associated with osteoporosis and fractures, this is an area which warrants further study [61].

### 4.4 Arthropathy

Joint bleeds/hemarthroses are typically associated with patients with hemophilia but may also occur in up to 25% of patients with VWD [12,13]. They are more common in patients with type 3 VWD, with a rate of 35% reported by the Willebrand in the Netherlands (WiN) study group [13].

Although hemarthroses are clearly a bleeding manifestation of VWD, they have wider implications on the health and wellbeing of patients with VWD. Occurrence of hemarthroses is associated with development of arthropathy with subsequent pain and disability. A comparison of 48 patients with VWD in the WiN study with .a history of joint bleeds (VWD-JB) and 48 without, reported a Hemophilia Joint Health Score of ≥10, indicative of the presence of arthropathy, in 40% of the VWD-JB group compared with 10% of the controls (*P* < .01); a Hemophilia Activity List score of <95, demonstrating the presence of functional restriction; in 67% of patients with VWD-JB vs 35% controls (*P* < .01); and a visual analog score of pain of >3, considered to be suggestive of clinically significant pain, in 35% of the VWD-JB group compared with 19% of controls (*P* = .07) [17]. Joint damage secondary to bleeding is also significantly associated with reduced quality of life (QoL), with increased chronic pain and the need for subsequent surgery [12,62].

Among patients with VWD and a history of bleeding rendering them eligible for factor prophylaxis, use of prophylaxis is strongly associated with reduced risk of arthropathy development. A post hoc analysis of the cost of VWD across Europe, a socioeconomic study (CVESS) analyzed outcomes in 229 patients with VWD receiving factor prophylaxis and 102 patients eligible for prophylaxis, but not receiving it. Patients were eligible for prophylaxis based on a history of recurrent bleeds, a critical-site organ bleed, or hospitalization due to bleeding. The group not receiving prophylaxis had significantly higher rates of chronically damaged joints (35.3% vs 19.2%; *P* = .0002), joints with reduced range of movement (37.3% vs 16.6%; *P* < .001), and chronic pain (2.4% vs 0.8%; *P* = .002) [63]. Careful assessment of a bleeding history and consideration of prophylactic treatment is therefore important not only in patients with severe VWD but also in those with moderate disease and a bleeding phenotype.

### 4.5 Cancer

The link between VWF and cancer biology is complex and incompletely understood. Plasma VWF levels are increased in patients with both solid tumors and hematological malignancies, with associated poor outcomes [64,65]. VWF levels impact risk of not only cancer-related thrombosis but also tumor cell apoptosis, angiogenesis, and metastasis [66]. Promotion of metastases by VWF appears to have platelet-dependent and independent mechanisms in a number of cancers. For example, blocking VWF in murine models of colonic carcinoma or melanoma impeded platelet-tumor interactions and development of metastatic disease [67]. However, other cancer models showed an increase in metastatic deposits in the absence of VWF, due to VWF-mediated tumor cell apoptosis [68,69]. Currently, there are no data to reliably suggest that patients with VWD are at increased or decreased risk of certain cancers, but this represents an area requiring further study.

## 5 Psychosocial Impact

VWD is a life-long condition. The ramification of chronic symptoms related to excessive bleeding can have a profound impact on QoL, mood, social interactions, and relationships, which may affect women disproportionately compared with men [70,71]. The European hemophilia network project requires that hemophilia comprehensive care centers have access to clinical psychology and social work support. A multidisciplinary approach to the care of patients with VWD is recommended [72]; however neither the American Society of Hematology/International Society of Thrombosis and Haemostasis (ISTH)/National Bleeding Disorders Foundation/World Federation of Hemophilia, or the British Society of Haematology guidelines directly discuss this aspect of care [3,73,74].

### 5.1 Depression/anxiety

Kirtava et al. [22] analyzed the incidence of self-reported depression in 102 women with VWD compared with 88 controls in their study for the CDC, finding no significant difference in the frequency of depression between cases and controls. However, other more recent studies have reported conflicting results. One group enrolled 272 females aged 9 to 25 years with either HMB or a bleeding disorder. Approximately 44% of patients had both conditions, and of the 51% of patients with a bleeding disorder, VWD accounted for 68% of cases. The incidences of depression and anxiety were 40% and 37% respectively, with the presence of HMB being significantly associated with both conditions in the group with a bleeding disorder [75]. Similar findings were observed in a study of >1000 adolescent females where 51% with HMB had depression compared with 24% without (*P* < .001) [76].

Another group investigated rates of depression and anxiety in 77 patients with VWD over the age of 12 years, using an 8-item patient health questionnaire for depression and the 7-item generalized anxiety disorder questionnaire. The instruments were administered prospectively as a part of a study. The rate of reported depression, defined by a score of ≥10 on the patient health questionnaire for depression instrument, was significantly higher among patients than reported by the general US population (63.6% vs 7%), with no difference by sex noted (females with VWD, 62.5%; males with VWD, 63.9%). However, although anxiety, defined as a score of ≥10 on generalized anxiety disorder 7, was also markedly more common in patients with VWD than that in the general population (53.9% vs 6.1%), it was present in approximately 15% more females with VWD than males with VWD (61.4% vs 46.7%), which did not meet statistical significance [77]. Based on the available data, there certainly seems to be a correlation between HMB in VWD and both depression and anxiety, particularly among adolescent girls.

As with some other conditions observed with increased frequency in these patients, the potential compounding impact of ID/IDA requires consideration. Iron is a cofactor of aromatic amino acid hydroxylase enzymes, the activity of which is required for the synthesis of neurotransmitters such as dopamine and serotonin, which play key roles in regulating mood and emotion [78]. Whether people with VWD are otherwise at increased risk of mood disorders requires further research.

### 5.2 Impact on sexual relationships

A patient survey study conducted by the European Haemophilia Consortium included 709 women with bleeding disorders across 32 countries, including 198 with VWD. Less than one-half of the VWD cohort reported that their bleeding disorder had no impact on their romantic and social life, with moderate-severe impact reported by approximately 40% [79]. A recent study in the Netherlands was undertaken specifically to assess the impact of bleeding on sexuality in VWD [80]; 549 patients over the age of 18 years with VWD were enrolled. Median age was 51 years, 63% were women, and types 1, 2, and 3 VWD accounted for 57%, 39%, and 4% of patients, respectively. Women were significantly more likely to report sexual restrictions than men (9.8% vs 3.5%; *P* < .01). One-third of women reported bleeding during sexual intercourse, and premenopausal women reported significantly greater restrictions on sexual activity than postmenopausal women, likely related to HMB (15.5% vs 5.2%; *P* = .01). HMB was identified as the most important factor affecting sexual activity in this study (odds ratio, 1.6; 95% CI, 1.12-2.46), again highlighting the huge impact this symptom has on women with VWD [80].

### 5.3 Quality of life

A systematic review assessing the impact of VWD on mental health analyzed results from 13 studies, reporting significant reductions in QoL, as measured by the health-related QoL tool, Short-Form (SF)-36. SF-36 analyzes 8 health domains, namely limitations in physical activities because of health problems, limitations in social activities because of physical or emotional problems, limitations in usual role activities because of physical health problems, bodily pain, general mental health, limitations in usual role activities because of emotional problems, vitality, and general health perceptions. Components of the SF-36 affected included vitality, relating to energy and fatigue, and mental health [81]. The Willebrand Study Health-related quality of life is a prospective observational trial in France, designed to assess QoL in 357 children and adults with VWD. Various cohorts have been reported, in particular those with type 2 VWD, accounting for 226 patients, of which 141 were female (101 adults and 40 children). Females had higher scores on the SF-36, indicating poorer QoL than males in both physical and mental components of the generic SF-36, as well as on the normalized global score specific to VWD, although it was not reported whether these differences reached statistical significance [82]. A large prospective study used the SF-36 to assess QoL in 509 patients with VWD (317 females). Compared with the general population, QoL was significantly reduced in the vitality domain for both sexes, with a significant association with bleeding severity observed [83]. Another study recruited patients with VWD from a tertiary care bleeding disorders clinic. Of the 102 patients enrolled, 80% had type 1 VWD and 78% were females. Compared with age- and sex-matched expected results from the general Canadian population, patients had significantly poorer QoL readings in physical and mental aspects of the SF-36 [84]. Given that the majority of patients in this analysis had mild VWD, the impact on QoL observed is concerning.

Patients aged ≥13 years registered with the Hamilton-Niagara Regional Haemophilia Program were invited to complete a health utilities index score 23S4E questionnaire; 28 patients with VWD completed the surveys, of whom 18 had type 1 VWD, 9 had type 2, 1 had type 3, and 64% were female. The age range was 13 to 73 years, with a mean and median age of 36 years. Compared with the general population, QoL was significantly reduced in the patients with VWD, particularly for the aspects of emotion, cognition, and pain. Notably, female participants had significantly worse scores than males, including in cognition. The authors postulated that this might be related to the deleterious impact of HMB on education, as only 5.5% of the women assessed had undertaken postgraduate education compared with 35% of the men. However, the subgroup from which these proportions were generated was not stipulated, and some of the patients assessed were below the age required for postgraduate education. The potential role of ID on cognition is an additional important consideration, which was not assessed specifically in this study [34,70].

## 6 Socioeconomic Consequences

VWD has a marked socioeconomic impact, from childhood through to old age. The negative impact of HMB on the ability of patients to attend school is well documented. The WiN study is a nationwide study of patients with VWD in the Netherlands performed from 2007 to 2009. Patients self-reported bleeding severity using the condensed Molecular and Clinical Markers for the Diagnosis and Management of type 1 VWD (MCMDM-1 VWD) score. Of 652 patients, the proportion with a low educational level increased with disease severity, from 36.8% in patients with type 1 VWD, to 40.2% in type 2 and 52.9% in type 3, compared with 36.4% in the general population. Notably, for female patients, the bleeding score for HMB was significantly associated with lower educational level. Number of days absent from school or work due to VWD also rose significantly from a median of 0 days for patients with type 1 and 2 VWD to 3 (IQR, 2-4) for children up to the age of 16 years and 4 (IQR, <0-10) for adults aged >16 years [85]. In a prospective study, 42 adolescent females between 12 and 19 years of age with an inherited bleeding disorder and HMB, 20 of whom had VWD, were invited to complete a QoL assessment during menstruation, prior to receiving treatment for their HMB and then again after treatment. Twenty-four patients completed the assessments. Prior to commencing therapy to reduce bleeding, 67% reported the need to reduce time spent on activities during menstruation, 63% felt that the types of activities they could participate in were limited, and 63% struggled with schoolwork. These rates fell to 8%, 4%, and 4% respectively after treatment [86]. These marked improvements with implementation of therapy highlight the impact that delayed diagnosis may have on females with VWD, and the need to improve education regarding HMB. Furthermore, another study of 30 women with VWD reported that nearly half of study subjects felt that their ability to work was reduced during menstruation [71], and women have also reported having financial difficulties in relation to losing their jobs due to their bleeding disorder [81]. It is clear that HMB can have a profound socioeconomic impact in female patients with VWD. In addition to this, ID and IDA negatively impact cognition, by decreasing memory, attention, and thought processing speed from early childhood into later life [36,87]. The vicious cycle of HMB leading to ID/IDA, impaired cognition and reduced engagement with academia, poorer opportunities for achievement, and subsequent impact on career advancement and financial stability significantly disadvantages in women with VWD compared with males and non-VWD peers.

Financial burden may be further exacerbated by parenthood, which also affects women more so than men. The motherhood penalty refers to the disproportionate workload carried by mothers compared with their male partners when it comes to childcare, often leading to reduced earnings and a lesser chance of workplace advancement [88,89]. This may be further compounded in inherited bleeding disorders like VWD, where affected children require additional care and women predominantly take on the carer role, often sacrificing their careers as a consequence [[90], [91], [92]].

The CVESS study was a retrospective study across Europe, which aimed to capture medical and non-medical costs of VWD over the course of 1 year. Data were collected on 708 adult participants, of whom 42% were females. Direct medical costs (eg. relating to hospitalization, surgical procedures, medical consultants, use of VWF concentrate, and other therapies) were higher for female patients than male patients (€59,012 vs €57,516 per year), which remained the case when cost of VWF concentrate was excluded (€10,402 vs €7,270 per year). Indirect costs considered loss of earnings and productivity, but unfortunately, the study did not provide subanalysis by sex [93]. We anticipate that the economic impact relating to loss of occupational earnings is likely to be more severe for women with VWD than that for men, with further research required to confirm this and infrastructural improvements desperately needed to support patients.

## 7 Conclusions

While bleeding is the primary symptom of concern caused by VWD, holistic care of patients requires more than mere management of bleeding episodes. Delay in implementing therapy for HMB in females with VWD due to lack of awareness of the issue by patients, family, carers, or medical practitioners, puts these patients at risk of harm that extends beyond ID and anemia. HMB can reduce time spent at school or work as well as limit social activities and interactions. VWD is a chronic illness, with associations with reduced QoL, depression, and anxiety, all of which may be more pronounced in women with this condition than men. Socioeconomic ramifications may extend into long-term impact on career progression and financial strain, exacerbated by the societal expectation for women to take on disproportionately greater caregiving roles and duties. In addition, increased menstrual bleeding may exacerbate other gynecologic issues such as ovarian cysts or endometrial polyps. Whether there is a true association between VWD and miscarriage and endometriosis certainly warrants further study, as does the cause of reduced BMD and how best to optimize bone health. Furthermore, current data do not provide any insight into whether VWD subtype may affect nonbleeding issues. While severe bleeding phenotypes may be anticipated to be associated with worse psychosocial and socioeconomic issues as well as ID, whether this has any bearing on other nonbleeding medical conditions is not known.

**Figure 2:**
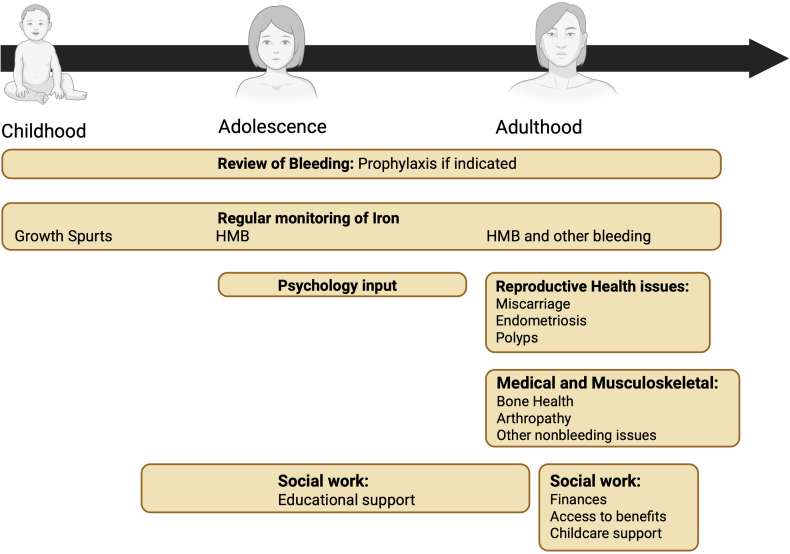
An approach to holistic care in women and girls with von Willebrand disease. HMB, heavy menstrual bleeding.

Implementation of integrated multidisciplinary management of patients with VWD, rather than merely considering bleeding episodes, is vital to improving the long-term health and wellbeing of these patients. A suggested approach is shown in Table 2 and Figure 2.

**Table 2:** An approach to holistic care in women and girls with von Willebrand disease.

Childhood	Adolescence	Adulthood
Medical review: Bleeding symptoms Assess iron stores (especially during growth spurts) Hemostatic prophylaxis if required	Medical review: Bleeding symptoms Assess iron stores (especially after onset of menarche) Hemostatic prophylaxis if required Management of HMB	Medical review: Bleeding symptoms Assess iron stores (frequency determined by degree of HMB) Hemostatic prophylaxis if required Management of HMB Consider bone health—review risk factors, eg, low body weight, smoking, and thyroid status Review reproductive issues and family planning
MDT input: Review educational progress Psychology support if required	MDT input: Review educational progress—secondary and higher education Psychology support if required	MDT input: Social work support—financial support, child care Psychology support if required
	Transition of care from pediatrics to adult care	

HMB, heavy menstrual bleeding; MDT, multi-disciplinary team.
